# Editorial: Cancer and cardiovascular diseases: common mechanisms and strategies of prevention

**DOI:** 10.3389/fphar.2026.2026318

**Published:** 2026-10-28

**Authors:** Paola Patrignani, Pietro Minuz, Dieter Steinhilber

**Affiliations:** 1 Department of Neuroscience, Imaging and Clinical Sciences, “G. d’Annunzio” University, Chieti, Italy; 2 Department of experimental oncology Mario Negri Institute for Pharmacological Research (IRCCS), Milan, Italy; 3 Department of Medicine, University of Verona, Verona, Italy; 4 Institute of Pharmaceutical Chemistry, Goethe University Frankfurt, Frankfurt, Germany

**Keywords:** cancer, cardiovascular disease, cholesterol, lipids, platelets, thrombosis

## Introduction

Cancer and cardiovascular diseases (CVD) are leading causes of morbidity and mortality worldwide. Although traditionally regarded as distinct disease entities, growing evidence indicates that they share key risk factors and molecular mechanisms, including chronic inflammation, oxidative stress, metabolic dysregulation, endothelial dysfunction, and altered platelet function. Moreover, their relationship is bidirectional: cardiovascular disease may accompany an increased risk of malignancy, while cancer and its treatment can substantially affect cardiovascular health. Advances in cancer therapy have improved survival but have also increased the clinical relevance of cardiovascular complications, making their prevention and management essential components of modern oncology.

This Research Topic, initiated in connection with the international meeting *Cancer and Cardiovascular Diseases: Common Mechanisms and Strategies of Prevention* held in July 2025, brings together 13 contributions addressing the molecular, pharmacological, and clinical interfaces between these diseases. The articles highlight three closely interconnected areas: common pathogenic mechanisms, cardiovascular complications of cancer and its treatment, and opportunities for integrated prevention and personalized therapeutic strategies.

## Inflammation and platelet activation as common pathogenic mechanisms

Chronic inflammation represents a central link between cancer and cardiovascular disease. Inflammatory mediators contribute to endothelial dysfunction, atherosclerosis, and adverse cardiac remodeling, while also influencing tumor initiation, progression, and interactions with the immune system. Importantly, inflammation is closely linked to platelet activation and lipid mediator biosynthesis, offering potential targets for interventions that address both conditions.

In this context, Di Berardino et al. discuss the contribution of platelet-derived thromboxane A_2_ (TXA_2_) to colorectal carcinogenesis and metastasis. Beyond its established role in thrombosis, TXA_2_ may promote tumor development through inflammatory signaling and suppression of antitumor immunity. These findings provide a mechanistic rationale for the potential anticancer effects of low-dose aspirin, particularly in genetically defined patient populations. However, the bleeding risks associated with antiplatelet therapy emphasize the need for careful patient selection and the development of safer pharmacological approaches.

The regulatory complexity of platelet function is further illustrated by Tai et al., who demonstrate that albumin inhibits platelet activation and thrombus formation through mechanisms involving its negatively charged surface residues and modulation of PKC/Akt signaling. These experimental findings identify an additional connection between plasma protein homeostasis and thrombosis, with potential implications for conditions associated with hypoalbuminemia, including advanced malignancy.

Inflammatory pathways also offer opportunities for cardiovascular intervention. In an observational study involving patients with ST-elevation myocardial infarction, Zhang et al. report that early treatment with the PCSK9 inhibitor evolocumab was associated with reduced IL-1β and IL-17A levels and improved left ventricular heart function in STEMI. Although causality remains to be established, these findings suggest that lipid-modifying interventions may extend beyond cholesterol reduction to involve inflammatory processes relevant to cardiovascular pathology.

## Thrombosis at the interface of cancer and cardiovascular disease

Cancer-associated thrombosis illustrates particularly well how malignancy, inflammation, endothelial injury, and coagulation interact. The management of thromboembolic complications is challenging because the prevention of recurrent thrombosis must be balanced against an increased risk of bleeding.

In a retrospective study of patients with colorectal cancer-associated venous thromboembolism, Liang et al. identify clinical predictors of recurrent thrombosis and bleeding, emphasizing the heterogeneity of thrombotic risk in this population. Complementing these findings, Xie et al. systematically review risk factors for catheter-related thrombosis in cancer patients. Their analysis distinguishes patient-, catheter-, and treatment-related determinants, underscoring the importance of individualized prevention strategies.

Together, these contributions highlight the limitations of uniform antithrombotic approaches and support more precise risk assessment that accounts for cancer characteristics, comorbidities, treatment exposure, and bleeding susceptibility.

## Cardiovascular consequences of cancer and anticancer therapies

The growing complexity of anticancer treatment has substantially expanded the spectrum of cardiovascular complications. These include myocardial dysfunction, arrhythmias, vascular injury, hypertension, and immune-mediated cardiac damage.

Liu et al. review the bidirectional relationship between breast cancer and CVD, emphasizing shared inflammatory, metabolic, and hormonal mechanisms alongside treatment-associated cardiotoxicity. Mędrek et al. focus specifically on radiation-induced heart disease in breast cancer patients and discuss strategies for prevention and cardioprotection.

The emergence of molecularly targeted therapies and immunotherapies has introduced additional challenges. Nardin et al. review the cardiovascular effects of targeted therapies for non-small-cell lung cancer, highlighting drug-specific toxicity profiles and approaches to cardiovascular surveillance and management. Similarly, Iram and Lebeche address the cardiovascular complications associated with modern immunotherapies, including immune checkpoint inhibitors and cellular immunotherapies.

An important additional dimension is the patient’s general physiological condition. Papasotiriou et al. discuss malnutrition, cachexia, and sarcopenia as potential susceptibility factors for cardiovascular complications in cancer patients. Their review highlights the need to consider systemic metabolic alterations and impaired physiological reserve when assessing cardiotoxicity risk.

Collectively, these articles underline that effective cancer treatment requires not only oncological efficacy but also the preservation of cardiovascular function through interdisciplinary management.

## Shared risk factors and opportunities for prevention

Identifying common risk factors may support preventive strategies that benefit both cancer and cardiovascular health. Using United Kingdom Biobank data, Geng et al. report an association between coronary heart disease and the subsequent development of colorectal polyps. Although such observational findings do not establish causality, they support further investigation into shared metabolic, inflammatory, and lifestyle-related determinants.

Lipid metabolism represents another important area of convergence. Hou et al. describe a nonlinear association between LDL cholesterol concentrations and mortality among cancer survivors. Their findings emphasize the complexity of interpreting lipid concentrations in patients with malignancy.

Potential pharmacological approaches targeting common pathways are also explored by Gao et al., whose systematic review and meta-analysis indicate beneficial effects of flavonoids on lipid metabolism and atherosclerotic plaque burden in ApoE-deficient mice. While these preclinical findings suggest anti-inflammatory and antioxidant mechanisms of interest, their clinical relevance requires further validation.

## Conclusion and future perspectives

Taken together, the contributions to this Research Topic demonstrate that cancer and cardiovascular diseases are interconnected through multiple biological and clinical mechanisms. Inflammation, platelet activation, thrombosis, and metabolic dysfunction emerge as particularly relevant areas of convergence, while the cardiovascular consequences of anticancer therapies represent a major challenge for contemporary clinical practice.

Nevertheless, identifying shared pathways does not necessarily mean targeting them will simultaneously protect against both diseases. Translating mechanistic findings into clinical benefit requires rigorous investigation, especially when interventions may produce competing effects on tumor progression, cardiovascular outcomes, and bleeding risk.

Future research should therefore focus on integrating molecular insights with prospective clinical studies, identifying reliable biomarkers for risk stratification, and developing preventive strategies adapted to individual patient characteristics. Ultimately, closer integration of oncology, cardiovascular medicine, and pharmacology will be essential to move beyond treating two separate diseases toward a more comprehensive, personalized approach to patient care.

